# Low Bioelectrical Impedance Phase Angle Is a Significant Risk Factor for Frailty

**DOI:** 10.1155/2019/6283153

**Published:** 2019-06-10

**Authors:** Satoshi Tanaka, Kei Ando, Kazuyoshi Kobayashi, Taisuke Seki, Takashi Hamada, Masaaki Machino, Kyotaro Ota, Masayoshi Morozumi, Shunsuke Kanbara, Sadayuki Ito, Naoki Ishiguro, Yukiharu Hasegawa, Shiro Imagama

**Affiliations:** ^1^Department of Orthopaedic Surgery, Nagoya University Graduate School of Medicine, Nagoya, Aichi, Japan; ^2^Department of Rehabilitation, Kansai University of Welfare Science, Osaka, Japan

## Abstract

The phase angle, which is measured via bioelectrical impedance analysis (BIA), is a clinically important bioimpedance parameter used for nutritional assessment and evaluating the risk of various diseases, such as locomotive syndrome (LS). It remains unclear if the phase angle is associated with frailty (fragile state of physical and mental health). We therefore examined this association in a large prospective sample. Of 1081 individuals receiving health checkups, 550 (male; 235, female; 365) were enrolled in this study. We applied the Japanese version of the Cardiovascular Health Study criteria to evaluate frailty and administered the 25-Item Geriatric Locomotive Function Scale to diagnose LS. The phase angle was measured via BIA. Multiple logistic regression analysis was used to evaluate the relationship between the phase angle and frailty. For all participants and for each sex, the phase angle was significantly lower among individuals with frailty. After controlling for age, sex, and body mass index, we found that a low phase angle was a significant risk factor of frailty. As a result of multiple regression analysis including other confounding factors, among male participants, a low phase angle was significantly related with both frailty (*P* = 0.015) and LS (*P* < 0.001), whereas among female participants, the low phase angle had a stronger association with frailty (*P* = 0.001) than with LS (*P* = 0.52). Our findings suggest that a low angle is a risk factor of frailty. Furthermore, among female participants, frailty has a stronger relation with the phase angle than does LS. Therefore, the phase angle may be considered a useful indicator of frailty that does not require lengthy or costly assessment.

## 1. Introduction

The elderly population is growing on a global scale, which is creating a series of worsening social problems that largely stem from age-related changes in the mind and body. For example, aging leads to an increase in the prevalence of adverse outcomes such as falls and a greater need for long-term care. Therefore, prolonging healthy life among elderly adults and shortening long-term care are important.

Recently, researchers have become increasingly interested in the concept of frailty among elderly people. Frailty refers to a fragile state of mind and body that leads to health problems and a reduced resistance to stress; it can influence body composition, physical function and physical activity, fatigue, psychophysiological state, and even social function [1]. The most well-known definition of frailty was conceived by Fried et al. [2], who suggest that frailty is reversible—that is, people can return to a healthy state from a state of frailty through appropriate intervention and support. To this end, numerous studies have explored what factors are associated with frailty.

Bioelectrical impedance analysis (BIA), which enables easy measurement of body composition, is now commonly used in general health checkups. BIA enables measurement of the differences in the electrical resistance of various tissues (e.g., fat, muscle, and bone) through application of a weak current to the body. Among the bioimpedance parameters measured with BIA, the phase angle is one of the most clinically important parameters. The phase angle is defined as the ratio of resistance (intracellular and extracellular resistance) to reactance (cell membrane-specific resistance) expressed as an angle. It is considered an indicator of cell membrane function and is commonly used for nutritional assessment and for assessment of the risk of various diseases [3, 4]. For example, locomotive syndrome (LS) and the progression of its risk stages are associated with a decrease in the phase angle [5, 6].

Currently, a limited number of studies have investigated whether frailty is associated with the phase angle [7, 8]. Therefore, in this study, we aimed to explore this association in a large-scale prospective sample of health-checkup participants in Japan. We also evaluated the association between the phase angle and risk of LS.

## 2. Materials and Methods

### 2.1. Participants

The study participants were volunteers who received health checkups supported by the local government of the town of Yakumo, Japan, in 2016-2017. This checkup has been done annually in this town since 1982. It consists of voluntary orthopedic and physical function examinations, as well as internal medical examinations and psychological tests [9–14]. We included all participants who underwent BIA and completed evaluations for frailty and LS. We excluded participants with a history of spine and limb joint surgery, severe knee injury, severe osteoarthritis, a history of fracture in the hip and spine, neurological disorders, severe mental illness, diabetes, and kidney or heart disease.

Among the 1,081 individuals who received a health checkup, 575 underwent BIA and completed the frailty and LS evaluations. Of these 575 participants, 25 were excluded due to the above-mentioned criteria. Therefore, 550 participants were ultimately included in the study.

The study protocol was approved by the ethics committee of human research and the institutional review board of our university. All participants provided written informed consent before participation. The study procedures were carried out in accordance with the principles of the Declaration of Helsinki.

### 2.2. The Phase Angle Measurement

The phase angle was measured using BIA. We used the InBody 770 BIA unit (InBody Co., Ltd., Seoul, Korea) to perform the BIA [4–6]. The accuracy of this device has been reported previously [15, 16]. It is capable of carrying out multifrequency measurements and can measure not only the whole-body phase angle, but also the segmental phase angles. In this study, we investigated using the whole-body phase angle at a 50 kHz frequency, which is the most common. Participants were asked to grasp the handles of the BIA device and stand on its platform, ensuring that both hands and the soles of their feet were in contact with a series of electrodes (two electrodes for each foot and hand). BIA measures whole-body impedance or the opposition of the body to alternating currents. It does this by measuring two components: resistance and reactance. The phase angle was calculated automatically by the BIA device from these two components according to the following formula: phase angle (°) = (reactance/resistance) × (180°/*π*). We had all participants undergo BIA on an empty stomach to avoid any confounding effect of diet.

### 2.3. Frailty Evaluation

We evaluated frailty by applying the Japanese version of the Cardiovascular Health Study (J-CHS) criteria [17], which were adapted from the original CHS criteria. The criteria include unintentional weight loss, fatigue, inactivity, poor grip strength, and slow walking speed. Unintentional weight loss was defined as a decrease in body weight of more than 2 kg in the past 6 months without any particular cause. Fatigue was defined as self-reported exhaustion and was assessed using the following question: “In the past 2 weeks, have you felt tired without a reason?” Activity level was evaluated using the following questions: “Do you engage in moderate levels of physical exercise or sports in an effort to maintain health?” “Do you engage in low levels of physical exercise in an effort to maintain health?” If participants answered “No” to both questions, they were considered to be inactive. Poor grip strength was defined as a grip strength of <26 kg in men and <18 kg in women based on the Asian Working Group for Sarcopenia criteria [18]. A slow walking speed was defined as a gait speed of <1.0 m/s [19]. In this study, participants with impairments in three or more of the five criteria were categorized into the frailty group, while those with fewer than three criteria were categorized into a nonfrailty group.

### 2.4. Locomotive Syndrome (LS) Evaluation

The 25-Item Geriatric Locomotive Function Scale (GLFS-25) is a self-administered questionnaire comprising 25 items. For our study, we used the Japanese version of the GLFS-25 (called “Locomo 25”). Each item is graded on a 5-point scale, from no impairment (0 points) to severe impairment (4 points) [20]. The sum of the item scores yields a total possible score ranging from 0 to 100, with higher scores indicating greater LS severity. The validity and reliability of this new measurement are satisfactory, with a cutoff score of ≥16 indicating LS; individuals with a GLFS-25 score of ≤15 were placed into the non-LS group [21].

### 2.5. Statistical Analysis

All continuous variables were expressed as means and standard deviations (SDs), while categorical variables were expressed as percentages. The Mann-Whitney* U* test was used to compare the two groups. A logistic regression analysis using the stepwise method was conducted to investigate the variables strongly related with frailty and LS. To examine the associations of the phase angle and some variables, a multiple regression analysis was conducted. A* P*-value of <0.05 with a confidence interval of 95% was considered significant in all analyses. The statistical analyses were conducted using SPSS Statistics 25.0 for Mac (IBM Corp., Armonk, NY, USA).

## 3. Results

Table 1 shows the demographics, the phase angle, and prevalence of frailty and LS for the total sample and by sex. Male participants were significantly older and had higher body mass index (BMI) and higher phase angle than the female participants. The prevalence of LS did not differ by sex, whereas the prevalence of frailty was significantly higher among female participants (3.0% for male participants, 14.3% for female participants).

Table 2 shows a comparison of the demographics according to frailty and LS status among all participants. Individuals in the frailty and LS groups were found to be significantly older and had significantly lower phase angles.  Table 3 shows the results of the same analysis by sex. Among male participants, the frailty and LS groups showed no differences in age or BMI but had significantly lower phase angles. For the female participants, those in the frailty and LS groups were significantly older and had lower phase angles. Furthermore, those in the frailty group had a significantly lower BMI.

Table 4 shows the results of the logistic regression analyses in which the presence or absence of frailty and LS were dependent variables and age, sex, BMI, and the phase angle were independent variables. After adjusting for age, sex, and BMI, a lower phase angle was found to be a significant risk factor for both frailty and LS.

Table 5 shows the results of investigating whether the phase angle was significantly associated with frailty and LS status by sex. A multiple regression analysis was performed with the phase angle as the dependent variable and frailty and LS status, age, and BMI as independent variables. Among male participants, frailty and LS were significantly related to a lower phase angle, but for female participants, only frailty was significant. In other words, a lower phase angle had a stronger association with frailty than with LS among female participants.

## 4. Discussion

To our knowledge, this study is perhaps the first to show that a lower phase angle is a risk factor of frailty in a prospective sample of Japanese adults attending health checkups. Furthermore, among female participants, frailty had a stronger association with a low phase angle than did LS.

As noted above, frailty has five main components: excessive weight loss, exhaustion, low activity, slowness, and weakness [2]. Researchers have therefore proposed unintentional weight loss, fatigue, inactivity, poor grip strength, and slow walking speed as quantifiable criteria for evaluating frailty [2]. This evaluation method was first used in the CHS and currently is the most widely used worldwide. Since we targeted Japanese individuals, we employed the modified J-CHS criteria [17]. However, it can be difficult to evaluate large numbers of participants easily in a short amount of time because measuring walking speed and grip strength and quantifying physical activity level requires considerable labor and facilities. To identify a somewhat easier measure of frailty, we focused on the phase angle, which can be readily quantified by BIA.

The phase angle has gained popularity in recent years because it is highly predictive of impaired clinical outcomes and mortality for various diseases [3–6]. The phase angle represents both the amount and quality of soft tissue, with a high phase angle reflecting higher cellularity and better cell membrane or cell function; thus, it is an indicator of cell health [22, 23]. In other words, a high phase angle suggests a healthy whole-body condition, whereas a low phase angle indicates a state of poor health. However, researchers have noted that the phase angle measurement is susceptible to age and sex [24]. To eliminate these effects, we conducted separate analyses by sex in this study and adjusted for age in the multivariate analysis. Furthermore, the relation between the phase angle and the frailty was investigated in more detail by comparing them with LS, which has been previously reported to be related to the phase angle.

We found that the phase angle was significantly lower among individuals with frailty and LS. The logistic regression analysis, even after adjusting for age, sex, and BMI, revealed that a low phase angle was a risk factor of frailty and LS. In the multiple regression analyses by sex, both frailty and LS in male participants, but only frailty in female participants, were significantly associated with the phase angle. The phase angle is known to correlate with various functional indicators [25] as well as nutritional status, muscle weakness, and sarcopenia [26, 27]. A lower phase angle is generally considered a prognostic predictor or an early predictor of various diseases [3, 28, 29]. We have similarly found that a low phase angle is associated with LS and osteoporosis [4–6]. All these suggested the correlation between low phase angle and frailty that we found.

In female participants, only frailty was significantly related with the phase angle, whereas LS was not. This finding might be attributed to the difference in the prevalence of frailty and LS between male and female participants. Among female participants, the prevalence of frailty and LS was about the same, which might have led to the detection of a significant difference between frailty and LS; by contrast, in male participants, the prevalence of frailty was lower than that of LS, which might have led to the lack of a significant difference. Another possible reason is that LS relates primarily to poor mobility, while frailty is a more multifaceted condition relating to muscle weakness, nutritional status, mental state, etc. The phase angle is considered an indicator of the general condition of the whole body, which means that a lower phase angle is a better indicator of frailty than of LS.

This study has several limitations. First, we targeted residents in rural areas, which differ widely in their living and working environments compared with urban areas. Therefore, the results were possibly biased. However, we did not examine patients visiting hospitals, but focused on residents who were interested in health; thus, our study has the advantage of targeting participants who are not likely to have a disproportionately low phase angle. Second, the results of BIA might differ depending on the manufacturer of the device. In the future, we should ensure standardized technology and cross-calibration of electrical resistance.

## 5. Conclusions

A low phase angle was not only a risk factor of LS, as previously reported, but also of frailty. The sex-based analysis showed that a low phase angle was significantly related to frailty and LS in men, whereas in women, the phase angle had a stronger association with frailty. We suggest that frailty, in addition to LS, should be further examined in individuals showing a low phase angle during health checkups. This might aid in the early detection of frailty and early intervention.

## Figures and Tables

**Table 1:** Demographic characteristics, phase angle, frailty, and LS prevalence data.

Variables	Total (N = 550)	Male (N = 235)	Female (N = 315)	*P*
Age (years)	64.5 (10.1)	66.3 (9.3)	63.1 (10.5)	*< 0.001 ∗∗∗*
BMI (kg/m^2^)	23.6 (3.5)	24.4 (3.3)	22.9 (3.4)	*< 0.001 ∗∗∗*
Phase angle (°)	5.1 (0.6)	5.5 (0.6)	4.8 (0.5)	*< 0.001 ∗∗∗*
Prevalence of frailty	9.5%	3.0%	14.3%	*< 0.001 ∗∗∗*
Prevalence of LS	12.0%	8.9%	14.3%	0.063

*∗∗∗* < 0.001, Mann-Whitney *U* test, Fisher's exact test.

Parameter values are shown as means (standard deviations) or numbers. Italic text indicates a significant difference.

BMI: body mass index; LS: locomotive syndrome.

**Table 2:** Comparison of demographics and phase angle by frailty and LS status among the total sample.

	Frailty			LS		
Variables	Non-frailty (N = 498)	Frailty (N = 52)	*P*	Non-LS (N = 484)	LS (N = 66)	*P*
Age (years)	64.2 (10.0)	67.9 (10.3)	*0.015 ∗*	64.1 (9.8)	67.7 (11.5)	*0.003 ∗∗*
Sex (male/female)	228/270	7/45	*< 0.001 ∗∗∗*	214/270	21/45	0.063
BMI (kg/m^2^)	23.7 (3.4)	21.8 (3.6)	*< 0.001 ∗∗∗*	23.5 (3.4)	23.9 (4.0)	0.51
Phase angle (°)	5.1 (0.6)	4.5 (0.6)	*< 0.001 ∗∗∗*	5.1 (0.6)	4.8 (0.7)	*< 0.001 ∗∗∗*

*∗* < 0.05, *∗∗* < 0.01, and *∗∗∗* < 0.001, Mann-Whitney *U* test, Fisher's exact test.

Parameter values are shown as means (standard deviations) or numbers. Italic text indicates a significant difference.

BMI: body mass index; LS: locomotive syndrome.

**Table 3:** Comparison of demographics and phase angle by frailty and LS status in both sexes.

	Male						Female					
	Frailty			LS			Frailty			LS		
Variables	Nonfrailty (N = 228)	Frailty (N = 7)	*P*	Non-LS (N = 214)	LS (N = 21)	*P*	Nonfrailty (N = 270)	Frailty (N = 45)	*P*	Non-LS (N = 270)	LS (N = 45)	*P*
Age (years)	64.2 (10.0)	67.9 (10.3)	0.46	66.0 (9.3)	70.0 (9.3)	0.071	62.4 (10.4)	67.7 (9.9)	*0.002 ∗∗*	62.6 (10.0)	66.6 (12.3)	*0.011 ∗*
BMI (kg/m^2^)	23.7 (3.4)	21.8 (3.6)	0.94	24.4 (3.1)	25.4 (5.0)	0.22	23.2 (3.4)	21.3 (3.3)	*0.001 ∗∗*	22.9 (3.5)	23.2 (3.2)	0.28
Phase angle (°)	5.1 (0.6)	4.5 (0.6)	*0.020 ∗*	5.6 (0.5)	5.1 (0.7)	*0.001 ∗∗*	4.8 (0.4)	4.4 (0.6)	*< 0.001 ∗∗∗*	4.8 (0.4)	4.5 (0.6)	*0.021 ∗*

*∗* < 0.05, *∗∗* < 0.01, and *∗∗∗* < 0.001, Mann-Whitney *U* test.

Parameter values are shown as means (standard deviations) or numbers. Italic text indicates a significant difference.

BMI: body mass index; LS: locomotive syndrome.

**Table 4:** Logistic regression analysis for the prediction of frailty and LS in the total sample.

	Frailty				LS			
Covariates	Coefficient (*β*)	Odds ratio	95% CI	*P*	Coefficient (*β*)	Odds ratio	95% CI	*P*
Phase angle (°)	−1.993	0.136	0.074-0.250	*< 0.001 ∗∗∗*	−1.165	0.312	0.192-0.506	*< 0.001 ∗∗∗*
BMI (kg/m^2^)				0.11	0.096	1.101	1.020-1.188	*0.014 ∗*
Age (years)				0.68				0.21
Sex (male)				0.20				0.52

*∗* < 0.05, *∗∗∗* < 0.001.

The dependent variable was LS or frailty. Independent variables were age, sex, BMI, and phase angle. Italic text indicates a significant difference.

CI: confidence interval; BMI: body mass index; and LS: locomotive syndrome.

**Table 5:** Summary of the multiple regression analysis for phase angle by sex.

	Male		Female	
Independent variable	Standardized partial regression coefficient (*β*)	*P*	Standardized partial regression coefficient (*β*)	*P*
Age (years)	−0.432	*< 0.001 ∗∗∗*	−0.387	*< 0.001 ∗∗∗*
BMI (kg/m^2^)	0.292	*< 0.001 ∗∗∗*	0.219	*< 0.001 ∗∗∗*
Frailty	−0.128	*0.015 ∗*	−0.177	*0.001 ∗∗*
LS	−0.189	*< 0.001 ∗∗∗*		0.52

*∗* < 0.05, *∗∗* < 0.01, and *∗∗∗* < 0.001.

The dependent variable was phase angle. The independent variables were age, BMI, LS, and frailty. Italic text indicates a significant difference.

BMI: body mass index; LS: locomotive syndrome.

## Data Availability

No data were used to support this study.
